# The Chicago Medical Society

**Published:** 1866-03

**Authors:** 


### PROCEEDINGS OF MEDICAL SOCIETIES.

### THE CHICAGO MEDICAL SOCIETY.

At the last meeting of this society, Dr. Ross presented, as pathological specimens, two kidneys, smaller than normal, having an average weight of $3\frac{3}{4}$ ounces each. Their surface was smooth, a portion of which appeared dark and congested. On peeling off the capsule, a portion of the renal tissue adhered to it, the texture of the kidney being softened. The venous vascularity was marked and irregular, presenting a mottled appearance. On a section, the cortical substance was diminished, not averaging over 3-5ths of an inch in thickness. The pyramids were distinct with a plain limiting line.

The Dr. stated that these were fine specimens of the *mottled fatty kidney.* The patient from whom they had been taken, had died comatose, in the County Hospital, March 1st. He had been admitted into the Hospital three weeks before, in the second week of his illness, with the usual marks of typhus fever. The marked symptoms were those of low fever, dry skin, frequent pulse, dry tongue, sordes, rash, constipation and delirium. There had been no dropsy, and no convulsions; evidently the affection of the kidneys had come on as a complication. How long they had been the seat of disease he did not know, there having been no symptoms directing attention to the kidneys; the urinary secretion had not been tested, but as the patient, previous to his sickness, had been in robust health, it is almost certain they had become affected sometime during the course of the fever. The specimens were interesting as showing the amount of fatty change which had developed in so short a time. A case is reported by Dr. Johnson in his work on the kidney, which came on during convalescence from typhus fever, which run its entire course in four weeks, to a fatal termination. The post mortem appearances of the kidneys are described as somewhat similar to the specimens presented by Dr. Ross, except the degeneration was probably much more extensive in the case of Dr. Johnson.

Dr. Ross also exhibited two kidneys taken from a phthisical patient brought to the Hospital in a moribund condition. The specimens were characterized by the absence of the line of demarkation between the cortical substance and the pyramids. The kidneys were slightly hypertrophied and softened, and presented evidences of fatty degeneration. In one of the specimens there was a large cyst filled with serum and urine.

A very interesting and quite rare case of malarial disease was reported by Dr. S. Wickersham. A boy, eight years of age, usually enjoying perfect health, had suffered for a week with daily attacks of torticollis. The attacks commenced about eleven o'clock A. M., and continued three or four hours, the head being drawn gradually to the left side, till it nearly reached the shoulder. The use of quinine for a few days entirely removed the affection. Two years ago, and again a year ago, the patient suffered every other day from similar attacks.

Dr. Charles G. Smith, reported a case of hæmaturia and one of dyspnœa, which will be found detailed in full on another page.

Dr. Holmes reported a ease of congenial opacity of the right cornea in an infant a month old. The family physician, Prof. Davis, and friends of the infant, observed the defect at birth, and stated that there had been no symptoms of inflammation, nor even of congestion of the eye. Nearly the whole cornea was densely opaque, there being a narrow rim at its upper and inner portion, which was quite translucent. The globe, as well as the cornea, was normal size, which is usually not the case where this deformity has been observed. The condition of the cornea seems to depend upon arrested development, and might possibly be considered as analogous to the opacity of the lens in congenital cataract. There is less probability that the opacity is the result of fœtal corneitis. Cases like the above are exceedingly rare.

Members of the society might attach whatever importance they chose to the following fact: In the third month of pregnancy, the mother had been much frightened by being informed that the right eye of one of her children had been "put out." Upon examining the child's eye, she found a piece of white tissue paper adhering to the cornea, over which the lids passed freely in winking. The children had been playing with a sheet of tissue paper attached to the end of a stick. By accident, the eye was struck by this paper, the corner of which coming in contact with the globe, was torn off and left adhering to the cornea. The mother stated that the eye, previous to the removal of the paper, appeared almost precisely like that of her infant.

An interesting discussion upon the subject of cholera followed the report of these cases. At some future time, we shall endeavor to present the readers of the JOURNAL with an abstract of what is now known regarding the nature and treatment of this disease. We would now merely commend two suggestions as worthy of consideration. Instead of chloroform to overcome spasm and increase the amount of blood in the brain, the attention of the society was called to the use of nitrous oxide gas. We might expect this agent not only to relieve spasm and vomiting, but also to supply oxygen freely to the blood. From the known effects of this gas in syncope, there are reasons for believing it may be of great benefit in nearly every stage of cholera.

A member described the following method of securing the action of mustard externally, in cases of cholera:

A dozen porous bricks, previously placed in boiling water till they have become thoroughly heated and saturated with moisture, should be covered with pulverized mustard of the best quality, and arranged on each side of the patient from head to foot. The patient should be then enveloped in thick blankets. The steam from the bricks carrying with it the active principle of the mustard, produces a most decided impression upon the whole surface of the skin. There is scarcely any method of applying mustard which will produce so rapid and thorough stimulation of the surface as this.

